# Nanoplastics measurements must have appropriate blanks

**DOI:** 10.1073/pnas.2411099121

**Published:** 2024-11-15

**Authors:** Dušan Materić

**Affiliations:** ^a^Helmholtz Centre for Environmental Research–UFZ, Department of Environmental Analytical Chemistry, Leipzig 04318, Germany

Qian et al. 2024 developed a data analyst platform for rapid Raman-based imaging of nanoplastics (1). They report micro- and nanoplastics concentrations of ~2.4 × 10^5^ per L of bottled water. In their quantification of micro- and nanoplastics exposure from the bottled water [(1), figure 5], they show that most of the plastic particles measured were Polyamide (PA—like nylon), polystyrene, and polyethylene terephthalate (PET), where PA was the dominate polymer type.

The author measured two types of blanks: Milli-Q water as complete *procedural blanks* and “*clean filters*.” However, when the authors analyzed the procedural blanks (Milli-Q water passed all the stages of filtration and analysis), they concluded that the procedural blanks were contaminated. They report in *SI Appendix*: “MilliQ water seems to have the same level of plastic contamination compared with bottled water measured (*SI Appendix*, Fig. S20*A*)”. Ignoring the observed procedural blank contamination, the authors decided to use the minimum contaminated not-procedural blanks to carry on their quantification, which is analytically incorrect: “To make sure the blank samples taken as reference are with minimize contamination, we eventually used Anodisc Al_2_O_3_ membrane filters as the blank for bottled water analysis this time.” (1), *SI Appendix*.

**Fig. 1.**
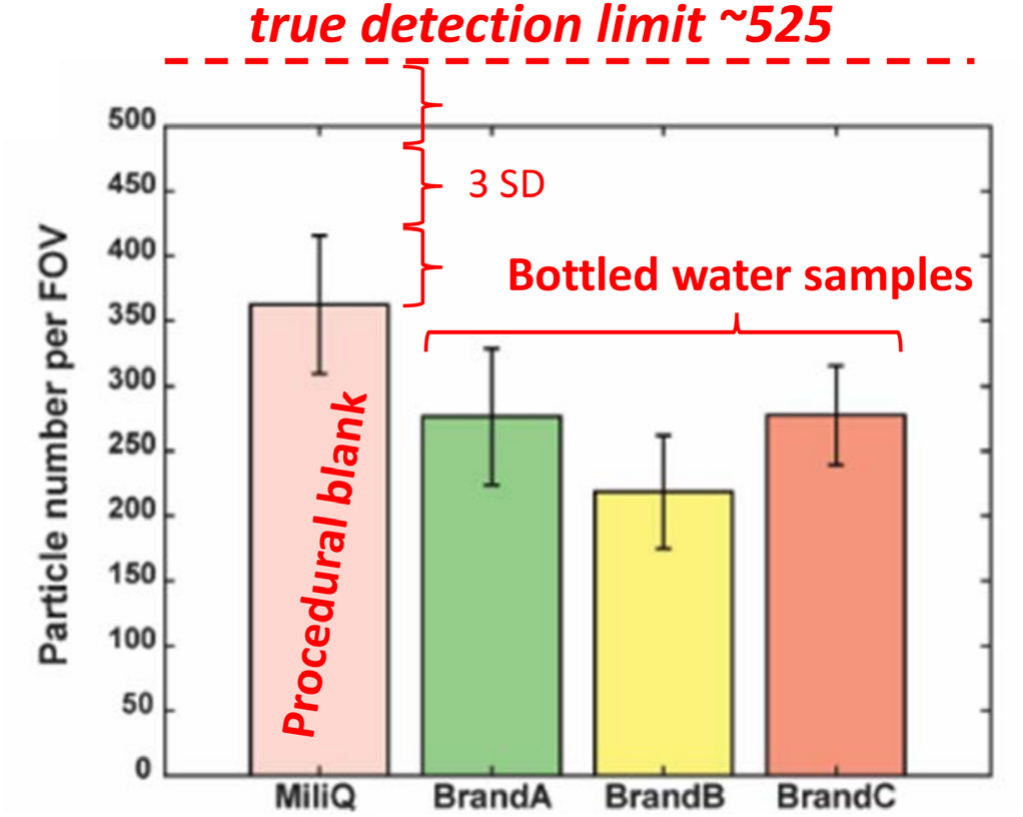
Estimating the true detection limit for particle number concentration based on the procedural blank sample reported by Qian et al. (1). Note that the measured concentration of micro/nanoplastics in every bottled water sample is below that of the procedural blank and far below the theoretical detection limit of +3 SD (or “3-sigma”) (2).

Any micro- or nanoplastics analysis [also any analytical measurement (2)] must use representative blanks to show the minimum quality control of the process (3). Accordingly, the appropriate blanks must cover all the analysis processes; thus, in the case of Qian et al. (1), Milli-Q water was a procedural blank that should have been used against the other bottled water samples (Fig. 1).

Using the clean, dry filters as the blank for water particulate matter (such as nanoplastics) ignores all the contamination which comes from the sample handling procedure (e.g., filtration of liters of water, subsampling process, use of aluminum foil covering, laboratory equipment contamination, nanoplastics loads in the air during sample preparation, plastics in chemicals/solvents, etc.) (4–7), which are known sources of contamination in each measurement (6). One needs to know those contamination levels for their particular experiment to calculate the detection limit and make any quantitative or qualitative conclusions (8). The authors had the opportunity to establish such a detection limit, but doing so risked having to acknowledge null results. By setting aside the plastic contamination measured in their procedural blanks, Qian et al. make any of their quantitative assessment of nanoplastics in bottled water fundamentally unreliable (with potentially harmful consequences for the field, effective policy-making, and public awareness).

Furthermore, the absence of appropriate quality control may have also compromised the qualitative aspect of this work, where the dominant nanoplastics polymer type in water from PET bottles was detected as PA, which, too, points to contamination or chemical identification issues.
